# Free flowing thought in bilinguals and monolinguals: How language experience and lexical properties influence word associations

**DOI:** 10.1371/journal.pone.0359889

**Published:** 2026-10-05

**Authors:** Matias Fernandez-Duque, Viorica Marian

**Affiliations:** 1 Department of Communication Sciences and Disorders, Northwestern University, Evanston, United States of America; 2 Department of Psychology, Northwestern University, Evanston, United States of America; Nihon Fukushi University: Nihon Fukushi Daigaku, JAPAN

## Abstract

Language shapes the organization of semantic memory and influences how individuals retrieve and combine concepts. The present study examined how language experience relates to associative thinking using Forward Flow, a metric that captures how far a thought sequence diverges semantically from an initial concept. Spanish-English bilinguals and English monolinguals completed a free association task in each of their respective languages. Greater vocabulary and language dominance predicted higher Forward Flow, indicating more semantically distant thought sequences. Among bilinguals, Forward Flow was higher in the dominant language than in the non-dominant language, suggesting that the conceptual associations bilinguals make depend on the language of retrieval. The associations participants generated were also influenced by idiosyncratic features of the languages they spoke, such as compounding, semantic ambiguity, and phonology. Our findings suggest that individual language experience and language-specific properties are linked to associative thinking, highlighting the role of language in the conceptual associations people make.

## Introduction

The ability to make associations and connections between seemingly unrelated concepts is a cornerstone of creativity. According to Mednick’s associative theory [1], highly creative people can retrieve remote concepts more easily and combine them in more creative ways. At the core of this associative ability is semantic memory, one’s knowledge of facts and concepts. Because access to semantic memory is often verbal, individual differences in language experience—such as speaking multiple languages—may shape how people form associations between concepts. Given that creativity differences are often observed between monolinguals and bilinguals [2], the current study investigated associative thinking as a potential mechanism underlying these effects. Specifically, we examined how language experience and linguistic features can influence concept retrieval during free associative thought.

Recent advances in computational modeling have bolstered researchers’ ability to quantify and study associative thinking. Particularly, distributional semantic modeling allows researchers to use large linguistic corpora to generate numerical representations of words [3]. In these models, word meanings are represented as coordinates within a “semantic space,” where words that are closer together in the vector space are closer in meaning than words that are further apart. The semantic similarity, or distance, of any two words can then be calculated, allowing researchers to quantitatively measure conceptual associations in natural language data [4].

One application of these computational techniques is Forward Flow, a metric that quantifies the semantic evolution of a thought sequence [5]. Forward Flow is typically applied to free association responses in which participants are given a starting seed word (e.g., *toaster*) and asked to generate a continuous sequence of words, producing each new word based on the one that came before (e.g., toaster → bread → sandwich → condiment). Forward Flow is then calculated by computing the semantic distance between the seed word and all subsequently generated words, providing an index of how far an individual’s ideas travel through semantic space. In this way, Forward Flow offers a computational means of capturing associative thinking, or the way in which individuals make conceptual associations. Importantly, metrics like Forward Flow have strengthened the associative theory of creativity as recent studies have found that Forward Flow predicts performance on lab-based creativity tasks, as well as membership in real-world creative fields like performance arts and entrepreneurship [5,6]. Because Forward Flow captures the associative processes that support creativity [4], it can serve as a powerful tool for investigating how individual differences, such as language experience, shape the foundations of creative thought.

Despite Forward Flow being an inherently linguistic measure that depends on language-based corpora and verbal responses, few studies have used it to investigate how language itself influences associative thinking. Language plays a key role in shaping how concepts are organized and accessed in semantic memory [7], and therefore likely plays a crucial role in associative thinking. Individual differences in language experience (e.g., bilingualism, vocabulary, dominance) modulate semantic memory organization [8] and word retrieval [9,10], which can impact the conceptual associations people make. For example, the specific associations bilinguals produce are shaped by their proficiency in the language at hand, among other factors [11]. Beyond individual experience, cross-linguistic variation in language-specific properties can also influence conceptual association patterns. For instance, languages have distinct lexical, phonological, idiomatic, and cultural features that could shape how concepts are associated within semantic memory. Bilinguals, who manage two linguistic systems that differ along many of these features, therefore represent an ideal test case for examining how both individual differences in language experience and cross-linguistic variation shape associative thinking.

Use of the Forward Flow metric with bilingual speakers has generally shown that word associations can vary considerably across a bilingual’s two languages. Fernández-Fontecha and Kenett [12] examined the associations between L2 fluency and creativity in Spanish (L1) students learning English (L2). Participants completed a creativity assessment and two semantic verbal fluency tasks (one in each language), and Forward Flow was calculated for each verbal fluency response sequence. Results showed Forward Flow was higher in Spanish (L1) than English (L2), indicating that participants provided more semantically distant responses in their more dominant, native language. In another recent study, Yang et al. [13] examined the relationship between L2 proficiency, Forward Flow, and creativity in a group of Chinese (L1) – English (L2) bilinguals. L2 vocabulary was positively correlated with Forward Flow, suggesting again that greater proficiency in a language facilitates the retrieval of more semantically distant concepts during associative thinking. Notably, both studies found that higher Forward Flow scores predicted higher creativity, extending Gray et al.’s [5] original monolingual findings to bilingual populations, and corroborating the claim that associative thinking underlies creative thought. However, neither study included monolingual participants, and Yang et al. [13] measured Forward Flow only in participants’ L2, without a parallel L1 measure. As a result, it remains unclear whether proficiency effects on associative thinking are specific to bilinguals, and how these effects differ across individuals’ two languages.

Although the use of the Forward Flow metric in bilingual studies is limited, extensive research suggests that dual-language experience shapes semantic memory and the conceptual associations bilinguals make. For example, studies have found that bilinguals rate unrelated word pairs as more semantically related than monolinguals [14,15], suggesting that concepts may be more closely associated in the bilingual mind. This may occur because words that are unrelated in one language can be related in form and/or meaning in a bilingual’s other language. Marian [16] proposes that such cross-linguistic overlap, which results in co-activation during language processing, forges links between concepts and strengthens their perceived similarity, in line with the “neurons that fire together, wire together” principle. Consistent with this view, bilinguals judge words as more semantically related when they share a translation equivalent [17,18] or are connected by a hidden false-cognate [19]. These findings suggest that distinct linguistic features from a bilingual’s two languages can influence the perceived similarity of concepts, which may alter how bilinguals retrieve and connect words during free associative thinking.

Building on this evidence, the present study examines associative thinking in English monolinguals and Spanish-English bilinguals. Using the Forward Flow measure, we trace the semantic trajectories of participants’ word sequences during a free association task. In addition to group-level comparisons (monolinguals vs. bilinguals), we investigate the role of language by analyzing how variation in language experience (e.g., vocabulary size, dominance) and linguistic features (e.g., word frequency, lexical ambiguity) shape conceptual associations. Although Forward Flow is not itself a measure of creativity, it captures a mechanism that supports creative thought: unstructured associative thinking. By examining associative thinking in bilinguals, along with the language-specific properties that shape it, we bring together two fields that have largely advanced independently: bilingual lexical processing and creativity research. In doing so, we aim to shed light on the complex relationship between bilingualism and creativity, which has to date produced mixed findings [2].

## Methods

### Participants

One-hundred and twenty Spanish-English bilinguals and 60 English monolinguals from the general population were recruited through Prolific and completed the study online using Pavlovia and Qualtrics. To qualify for participation on Prolific, participants had to indicate they resided in the United States, were between 18 and 35 years old, were free of any known language disorders or cognitive impairments, and identified as either English monolinguals or Spanish-English bilinguals. To further ensure eligibility for the study, participants completed a pre-screen questionnaire in Qualtrics prior to the start of the experiment. Inclusionary criteria for bilingual participants consisted of high self-rated proficiency in both English and Spanish (≥ 8 of 10), whereas inclusionary criteria for monolingual participants consisted of high self-rated proficiency in English (≥ 8 of 10) and little to no experience with other languages (self-rated proficiency of ≤ 2 of 10 in other languages). In addition to the experimental tasks, participants completed the Language Experience and Proficiency Questionnaire (LEAP-Q) [20], an objective vocabulary test (Multilingual Naming Test Sprint 2.0 [21]), and a nonverbal IQ test (matrix reasoning subtest of the Wechsler Abbreviated Scale of Intelligence, Second Edition, WASI-II [22]; see Table 1). Compared to bilinguals, monolinguals reported an earlier age of acquisition (AoA) in English, *t*(167.49) = 10.86, *p* < .001, had greater English exposure, *t*(132.44) = −23.35, *p* < .001, and scored higher on the English MINT Sprint 2.0, *t*(129.42) = −4.79, *p* < .001. As a group, bilinguals showed greater English than Spanish experience, with a later AoA, *t*(115) = 7.64, *p* < .001, greater exposure, *t*(116) = 2.93, *p* = .004, and higher MINT Sprin*t* 2.0 scores, *t*(119) = 5.49, *p* < .001. Both groups were comparable in age, *t*(109.51) = −1.98, *p* = .051, gender, *χ²*(2) = 0.45, *p* = .80, years of education, *t*(92.68) = 0.49, *p* = .626, and nonverbal IQ, *t*(128.65) = −0.84, *p* = .40. Informed consent was ob*t*ained online from all participan*t*s in accordance wi*t*h the Northwestern University Institutional Review Board (STU00023477). Participants were recruited between June 15, 2024, and September 2, 2024.

**Table 1:** Participant demographics.

Measure	Spanish-English Bilinguals	English Monolinguals	*p*-value
N	120	60	
Age	27.1 (4.4)	28.6 (4.8)	n.s.
Gender	71 F, 45 M, 4 O	33 F, 26 M, 1 O	n.s.
Years of Education (LEAP-Q)	15.2 (2.3)	15.0 (3.1)	n.s.
English AoA (LEAP-Q)	4.8 (3.2)	1.1 (1.3)	***
Spanish AoA (LEAP-Q)	1.9 (2.8)	-	-
English Exposure % (LEAP-Q)	53.9 (20.3)	99.2 (4.0)	***
Spanish Exposure % (LEAP-Q)	43.2 (19.2)	-	-
English Vocabulary (MINT Accuracy)	60.5 (9.5)	67.2 (8.6)	***
Spanish Vocabulary (MINT Accuracy)	50.7 (14.2)	-	-
Nonverbal IQ (Matrix Reasoning; WASI-II)	52.1 (7.8)	53.0 (7.1)	n.s.

*Note.* F = female; M = male; O = Other/NA; AoA = Age of Acquisition; LEAP-Q = Language Experience & Proficiency Questionnaire [20]; MINT = Multilingual Naming Test Sprint 2.0 [21]; WASI-II = Wechsler Abbreviated Scale of Intelligence, Second Edition [22]. Values represent means with standard deviations in parentheses. n.s. = *p* > .05, ***p < .001.

### Measures

**Forward Flow.** The Forward Flow task was administered following the procedure described in the original validation study by Gray et al. [5]. On each trial, participants received one of four seed words (*table*, *paper*, *candle*, *toaster*) and were asked to fill in 19 blanks with the first word that came to mind from the previous word, for a total of 20 words per trial (1 seed word + 19 generated words). Each trial was untimed and participants could proceed once they filled in the 19 blanks. The four trials were randomly split into two blocks. Bilinguals first completed two trials in one language and the other two trials in the other language (randomized order). Monolinguals completed all four Forward Flow trials in English. To situate our findings within the existing literature, the four seed words were chosen from the six seed words (table, paper, candle, toaster, bear, snow) used in Gray et al. [5]. Four seed words were chosen to minimize overall testing time and participant fatigue, and the seed words “bear” and “snow” were excluded to avoid confounds related to environmental or cultural exposure to these concepts in Spanish-English bilinguals (e.g., climate-dependent familiarity with snow, regionally dependent exposure to bears).

A Forward Flow score for each trial was calculated following the computational approach and formula developed by Gray et al. [5]. Using the R package *SemanticDistance* [23]*,* we calculated the average semantic distance (GloVe; [24]) between each generated word and all words preceding it, yielding an *instantaneous forward flow* (IFF) metric for each of the 19 generated words. The final Forward Flow score for each trial is the grand average of these distances, representing the total forward flow of each 19-word thought sequence. Forward Flow scores can theoretically range from 0 to 1, although in practice, GloVe-based Forward Flow scores tend to range from approximately 0.68 to 0.94 [6]. To use the same word embeddings across languages, Spanish responses were translated automatically into English using Google Translate and checked manually by a Spanish-English bilingual researcher.

**Multilingual Naming Test Sprint 2.0 (MINT;** [21]**).** The MINT is a picture naming test of productive vocabulary that is predictive of language proficiency [25,26]. All participants completed the MINT in English, and bilinguals also completed it in Spanish. For each language, participants were shown an array of 80 objects (10 pictures per row) that increased in naming difficulty. Participants began by pressing a button to start the recording, named as many pictures as possible in one pass, and then pressed the button again to end the recording. Verbal responses were manually transcribed, and MINT scores were calculated based on the number of correct responses (i.e., accuracy). Median completion time was 125 seconds (*Mean* = 136, SD = 47.5) for English and 146 seconds (*Mean* = 160, *SD* = 55.1) for Spanish. Monolinguals (*Median* = 112 seconds, *Mean* = 118, *SD* = 27.3) completed the English MINT faster than bilinguals (*Median* = 135 seconds, *Mean* = 145, *SD* = 52.6, *t*(164.44) = 4.47, *p* < .001), while bilinguals completed the English MINT faster than the Spanish MINT (*Median* = 146 seconds, *Mean* = 160, *SD* = 55.1), *t*(110) = −2.08, *p* = .040.

**Wechsler Abbreviated Scale of Intelligence – Second Edition (WASI-II;** [22]**).** Nonverbal IQ was assessed in between experimental blocks using the matrix reasoning subtest of the WASI-II. Participants were presented with incomplete visual patterns and had to select the piece (out of 5 options) that correctly completed the pattern. A total of 27 trials were presented. Raw scores were obtained and converted to standard T-scores (*μ* = 50, *SD* = 10) using the WASI-II age-based norming table.

### Procedure

The current report analyzed data from a broader project that included multiple experimental tasks [27]. Below is a detailed description of the procedure followed for the tasks (Forward Flow, WASI) and language measures (MINT, LEAP-Q) analyzed in this manuscript.

After providing consent and passing screening criteria, participants completed an initial block including two Forward Flow trials, followed by the matrix reasoning subtest (WASI-II), and then a second block with the remaining two Forward Flow trials. For bilingual participants, the order of languages for each block was randomized; monolinguals completed both blocks in English. After the second Forward Flow block, participants completed the MINT in each of their languages and the LEAP-Q in English. Upon completion, participants were debriefed and compensated monetarily for their time through the Prolific platform.

### Data analysis

To verify the quality of the data collected, all responses were manually reviewed prior to analysis. Only one bilingual participant’s Spanish block showed evidence of not following instructions (repeated words) and was excluded from analyses. Additionally, eighteen missing MINT scores (6% of scores) were imputed using the *mice* R package [28], an approach shown to be better than deletion or mean replacement methods [29,30].

To examine the effects of bilingualism on continuous associative thought, we first used linear mixed-effects regressions with each Forward Flow score (four per participant) as the dependent variable. The models were constructed using the R *lmer* function from the *lme4* package [31], and the significance of fixed effects was assessed through Likelihood Ratio Tests (LRTs) using the *mixed* function from the *afex* package [32]. Follow-up tests of simple effects were conducted with the *emmeans* and *emtrends* functions from the *emmeans* package [33].

Bilinguals and monolinguals were compared using a three-level categorical variable of Language Group that included the language of the task: monolinguals (in English), bilinguals (in English), bilinguals (in Spanish). Language Group was Helmert-coded to create two contrasts: (1) Monolinguals_Eng_ (+0.67) vs. Bilinguals_Spa_ (−0.33) and Bilinguals_Eng_ (−0.33), and (2) Bilinguals_Spa_ (+0.5) vs. Bilinguals_Eng_ (−0.5). We also used a categorical variable of Task Language Dominance to compare participants’ Forward Flow performance in their dominant (L_Dom_) or non-dominant language (L_NonDom_). For each participant, the dominant language was defined as the language with the higher MINT score. The Task Language Dominance variable was then coded as L_Dom_ if the language of the task matched the participant’s dominant language and L_NonDom_ if it did not. Task Language Dominance was then Helmert-coded with the following contrasts: (1) Monolinguals L_Dom_ (+0.67) vs. Bilinguals L_Dom_ (−0.33) and Bilinguals L_NonDom_ (−0.33) and (2) Bilinguals L_NonDom_ (+0.5) vs. Bilinguals L_Dom_ (−0.5).

We first examined participants’ total Forward Flow score for each trial. The final models for Forward Flow included fixed effects of Language Group or Task Language Dominance, English Vocabulary (English MINT z-scores), and their interaction. The model also included Seed Word and average Word Frequency (Log10 z-scores) of generated words as covariates, along with their interaction, as well as a by-participant random slope for Trial. Word frequency norms were retrieved from Brysbaert and New [34] for English and Cuetos et al. [35] for Spanish. The final model was selected based on the maximal random-effects structure justified by the design that successfully converged, and the best-fitting model was chosen using the Akaike Information Criterion. Age, Nonverbal IQ, and Forward Flow completion time were considered as covariates but not ultimately included as they did not improve model fit.

Our next analysis examined participants’ forward flow trajectory throughout the course of each trial with *instantaneous forward flow* (IFF), which captures the average semantic distance between each generated word and all preceding words. To model IFF throughout a trial, we used a generalized additive model (GAM) implemented with the *gam* function from the *mgcv* R package [36]. Our GAM used the same fixed effects and interactions as the previous analysis (Language Group × English Vocabulary and Seed Word × Word Frequency) but added smooth terms of Word Position to capture nonlinear IFF trajectories. Smooths were specified for positions 2–20 separately for each Language Group × Seed Word combination (*k* = 6), and a subject-level random effect was included via a random-effects smooth.

Given the differences our IFF analysis revealed during the beginning of the Forward Flow task (i.e., words 2–6), we used a binomial generalized linear model (GLM) to examine the probability of specific words being used during the first five responses in each trial. To minimize zeros in our model, we included all unique responses that appeared at least twice during the first five generated responses. We then calculated the frequency of each response and modeled the probability of it being generated for each seed word and language group. Estimated marginal means (*emmeans* R package; [33]) were then used to derive and compare each word’s predicted probability by seed and group.

## Results

### Forward flow

We first examined Forward Flow (FF) across language groups and seed words. There was a significant effect of Language Group (LRT: *χ*^*2*^(2) = 25.28, *p* < .001; see Table 2 and Fig 1A) suggesting Forward Flow differed between monolinguals and bilinguals. Estimated marginal means showed this difference was driven by bilinguals’ scores in Spanish (M = 0.689, 95% CI [0.682, 0.695]), which were significantly lower than bilinguals’ English scores (M = 0.705, 95% CI [0.699, 0.711]; *β* = 0.016, *t*(290) = 4.51, *p* < .001) and monolinguals’ English scores (M = 0.708, 95% CI [0.700, 0.717]; *β* = 0.020, *t*(228) = 3.71, *p* < .001). There was no significant difference in English scores be*t*ween monolinguals and bilinguals (*β* = 0.003, *SE* = 0.005, *t*(223) = 0.63, *p* = .804). There was a significant Language Group × English Vocabulary interac*t*ion (*χ*^*2*^(2) = 7.47, *p* = .024) where higher English Vocabulary predicted higher Forward Flow scores for monolinguals (*β* = 0.009, *SE* = 0.004, *t*(183) = 2.14, *p* = .033), but not for bilinguals (*p*s > .10).

**Table 2:** Likelihood ratio tests for fixed effects predicting forward flow.

Effect	df	*χ* ^*2*^	*p*-value	
Language Group	2	25.28	< .001	***
English Vocabulary	1	0.95	.329	
Seed Word	3	33.11	< .001	***
Word Frequency	1	290.06	< .001	***
Language Group × English Vocabulary	2	7.47	.024	*
Word Frequency × Seed Word	3	26.16	< .001	***

Model: Forward Flow ~ LangGroup*EngVocab + SeedWord*WordFreq + (1 + Trial | Subject)

**Fig 1:**
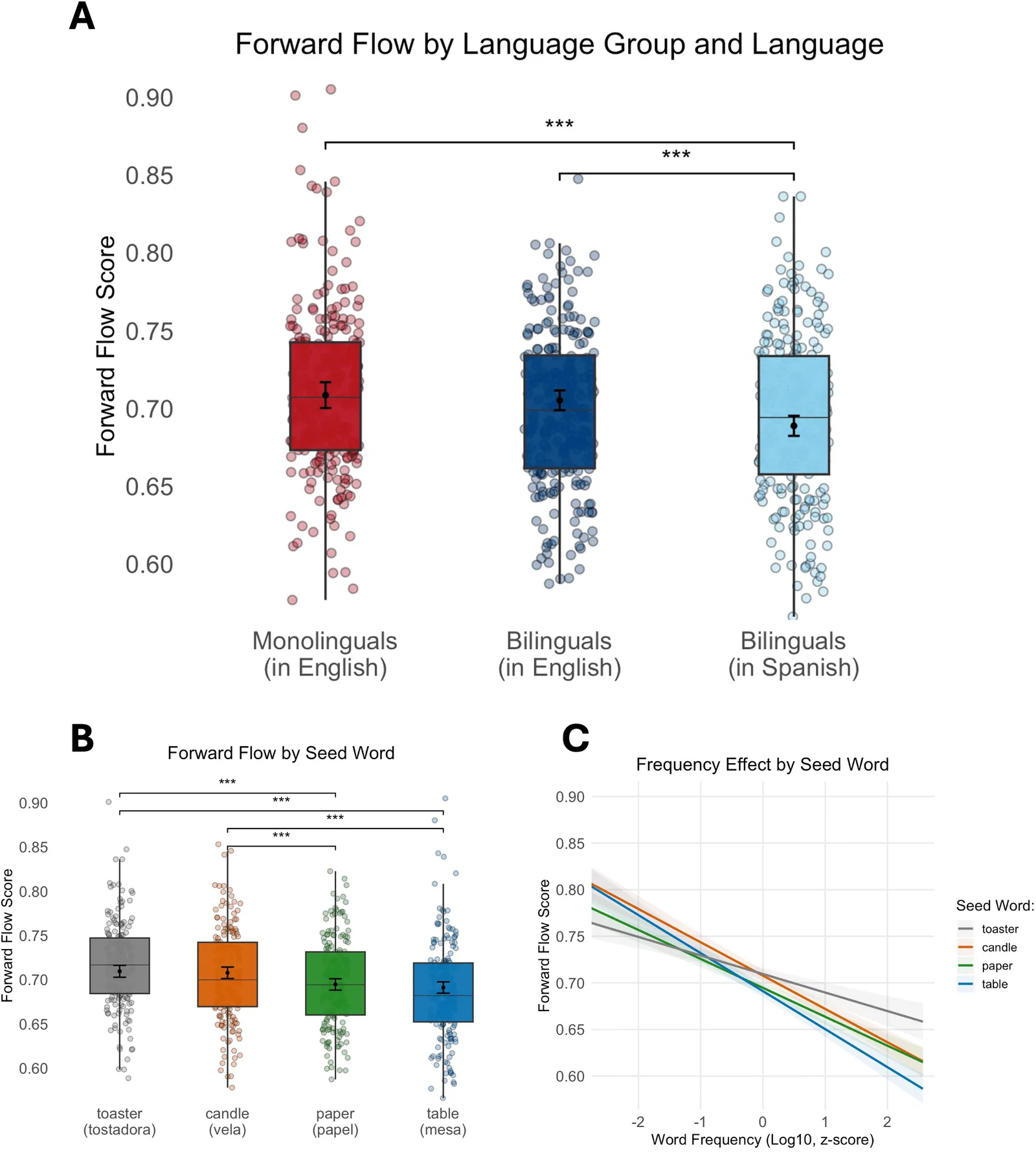
Forward Flow scores. (A) Monolinguals and bilinguals had higher Forward Flow scores in English than bilinguals did in Spanish. (B) Forward Flow scores varied by seed word: lower frequency seed words (e.g., *toaster* and *candle*) elicited higher Forward Flow than higher frequency seed words (e.g., *paper* and *table*). (C) The lexical frequency of words generated by participants strongly predicted Forward Flow scores, with lower-frequency words resulting in higher Forward Flow scores.

We also found a significant Word Frequency × Seed Word interaction (*χ*^*2*^(3) = 26.16, *p* < .001) indicating that the effect of frequency on Forward Flow varied by seed word (Fig 1C). Overall, using lower frequency words was associated with significantly higher Forward Flow scores (Word Frequency: *χ*^*2*^(1) = 290.06, *p* < .001) and Forward Flow scores varied significantly by Seed Word (*χ*^*2*^(3) = 33.11, *p* < .001; Fig 1B). Estimated marginal means showed that participants achieved higher Forward Flow when they started with lower frequency seed words like *toaster* (M = 0.709, 95% CI [0.703, 0.716]) and *candle* (M = 0.708, 95% CI [0.701, 0.714]) compared to when they started with higher frequency words like *paper* (M = 0.695, 95% CI [0.688, 0.701]), and *table* (M = 0.691, 95% CI [0.685, 0.698]).

We also examined Forward Flow scores across groups and participants’ dominance (Dominant vs. Non-Dominant) in the language of the task. The pattern of results mirrored the previous analysis, as Task Language Dominance, Word Frequency, and Seed Word were all significant predictors of Forward Flow scores (LRTs: *p*s < .01; see Table S1 in S1 File). Estimated marginal means showed the effect of Task Language Dominance was driven by bilinguals’ Forward Flow scores in their non-dominant language (M = 0.691, 95% CI [0.684, 0.697]), which were significantly lower than Forward Flow scores in their dominant language (M = 0.704, 95% CI [0.697, 0.710]; *β* = 0.013, *t*(296) = 3.49, *p* = .002), indica*t*ing bilinguals traveled farther semantically in their more dominant language. The difference in Forward Flow scores between bilinguals’ dominant language and monolinguals’ English was not significant (*β* = 0.005, SE = 0.005, *t*(226) = 0.92, *p* = .625).

### Instantaneous Forward Flow

To follow up on the effects of Word Frequency and Seed Word, we analyzed the semantic trajectory of generated words over the course of the Forward Flow task. To do so, we calculated *Instantaneous Forward Flow* (IFF) for each of the 19 generated words, which is the average semantic distance from any given word to all preceding words.

We used estimated marginal means to compare IFF at specific points of the free association task. We first examined IFF for the first generated word (i.e., the semantic distance between the seed word and second word). The seed words *toaster* (M = 0.649, 95% CI [0.640, 0.658]) and *candle* (M = 0.626, 95% CI [0.616, 0.636]) elicited a more semantically distant first word (i.e., higher IFF) than the seed words *table* (M = 0.539, 95% CI [0.528, 0.549]) and *paper* (M = 0.509, 95% CI [0.498, 0.520]; *p*s < .001; see Table S2 in S1 File for pairwise comparisons), possibly because of their lower frequency. This difference in IFF between seed words continued for the first five generated words, after which IFF became comparable across groups and seed words (Fig 2). Further analyses revealed group differences as well. For the seed word *candle*, bilinguals’ first generated word was significantly more distant than that of monolinguals. This difference was significant whether bilinguals responded in English (M = 0.635, 95% CI [0.620, 0.649]) or Spanish (M = 0.647, 95% CI [0.633, 0.661]), compared to monolinguals (M = 0.597, 95% CI [0.578, 0.616]; *p*s < .01).

**Fig 2:**
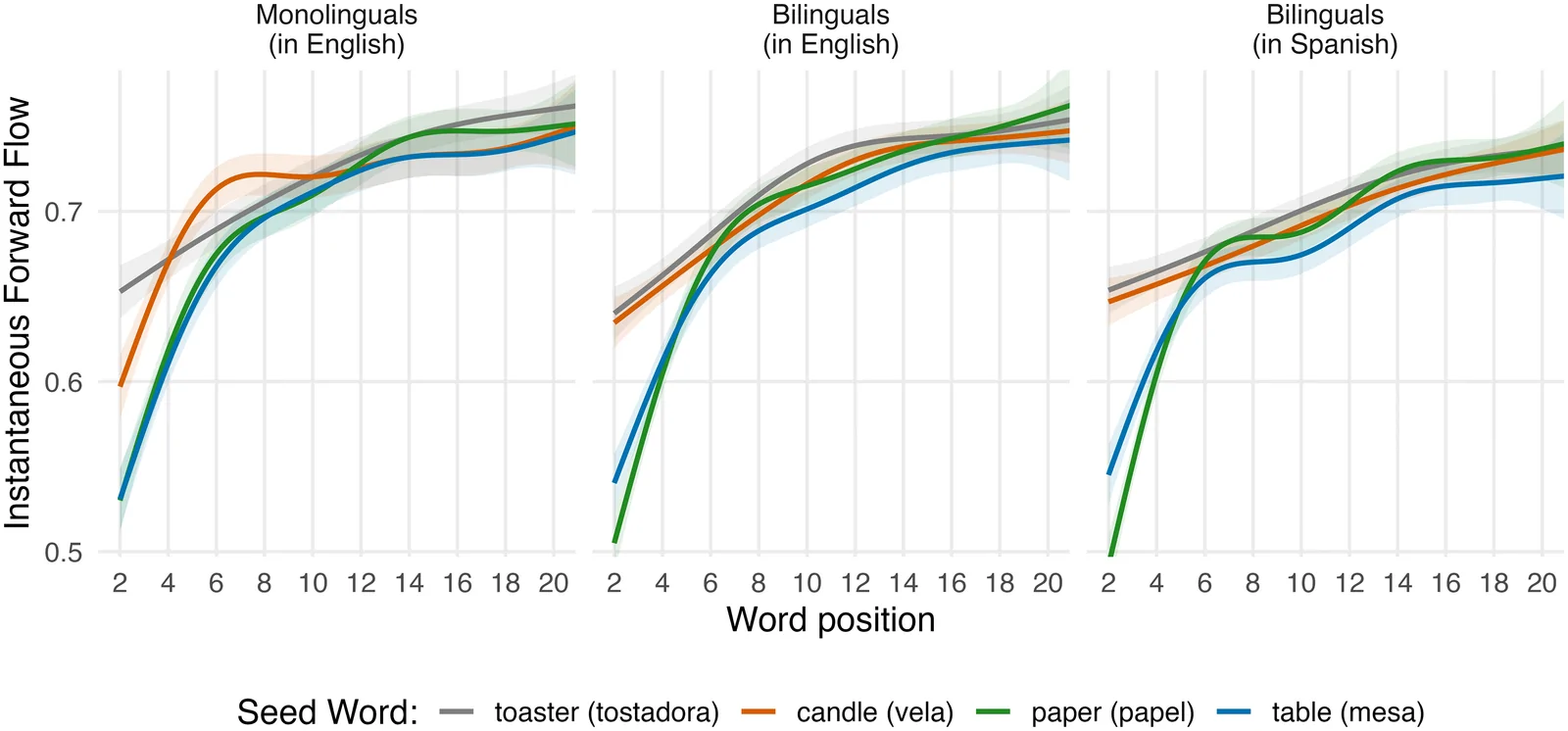
Instantaneous Forward Flow (IFF) across language groups and task language. Differences in IFF trajectories were largest in the beginning of the task (first five words). Lower frequency words (*toaster* in grey, *candle* in orange) elicited more distant initial words and flatter subsequent trajectories, and higher frequency words (*paper* in green, *table* in blue) elicited less distant initial words and steeper subsequent slopes.

Given the differences seen in the beginning of the task, we next examined the slope of IFF for the first five generated words. There were significant differences in IFF slope across items, with higher frequency seed words (*table* and *paper*) showing significantly steeper slopes than lower frequency words (*toaster* and *candle*; pairwise comparisons of estimated marginal trends; *p*s < .001). When examining both variables Language Group and Seed Word together, we again found that differences in slope between language groups were strongest for the seed word *candle*: monolinguals showed a significantly steeper slope (*β* = 0.028, 95% CI [0.023, 0.033]) than bilinguals in English (*β* = 0.011, 95% CI [0.007, 0.015], *p* < .001) and bilinguals in Spanish (*β* = 0.005, 95% CI [0.002, 0.008], *p* < .001; see Table S3 in S1 File).

Altogether, we found differences in IFF across language groups and seed words, especially in the beginning (words 2–6) of the Forward Flow task. The lexical frequency of seed words was associated with different semantic trajectories, with lower frequency words (*toaster*, *candle*) prompting more distant initial words and flatter subsequent trajectories, and higher frequency words (*paper*, *table*) prompting less distant initial words and steeper subsequent slopes.

### Words generated

Our final analysis examined the specific words generated during the Forward Flow task, focusing again on the first five generated words as a follow-up to the previous analyses. We found distinct patterns across both group (Monolingual vs. Bilingual) and language (English vs. Spanish; see Fig 3).

**Fig 3:**
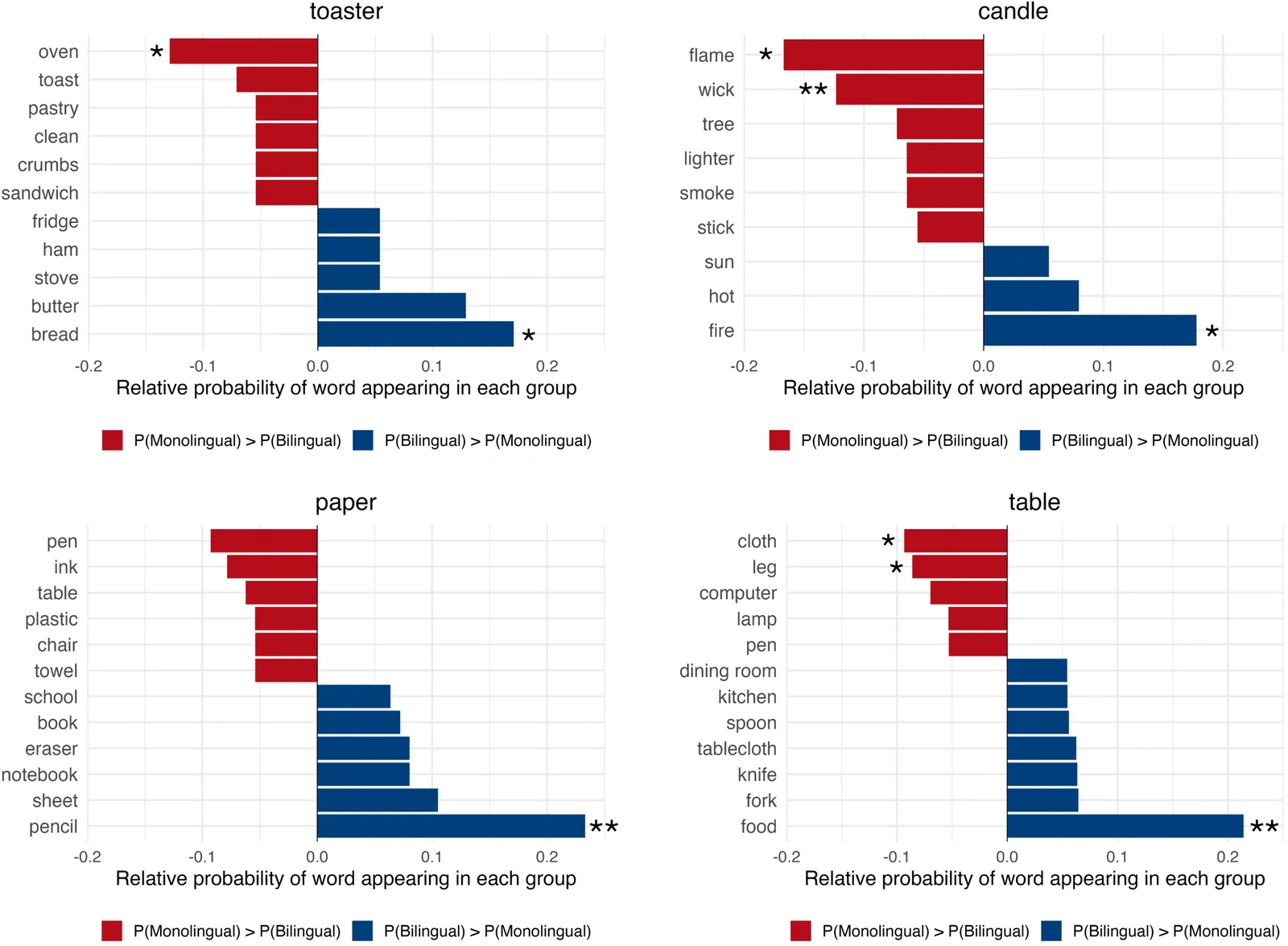
Distinctive words generated by monolinguals and bilinguals. In red are words that were more likely to be generated by monolinguals than bilinguals, and in blue are words that were more likely to be generated by bilinguals than monolinguals. Monolinguals were more likely to generate compound-based responses common in English (e.g., toaster *oven*, table*cloth*) and used more specific, lower-frequency words (e.g., flame, wick, lighter, smoke). Bilinguals were more likely to use broader, higher-frequency words (e.g., fire, food). Only the most distinctive words (absolute relative probability > .05) are shown. * *p* < .05; ** *p* < .01.

Mirroring previous analyses, the lower frequency seed words (*candle*, *toaster*) elicited the clearest group differences. Monolinguals were more likely to generate specific, lower-frequency words (e.g., *candle*: flame, wick, lighter, smoke; *toaster*: pastry, crumbs), whereas bilinguals were more likely to generate broader, higher-frequency words (e.g., *candle*: fire, hot; *toaster*: bread, butter). Across seed words, monolinguals were also more likely than bilinguals to use compound-based responses, often generating the second element of common English compounds (e.g., toaster *oven*, candle *stick*, paper *towel*, table *cloth*).

Certain language-specific collocations—series of words or terms that often co-occur—also appeared to guide responses in the task. For example, the seed word *paper* was more likely to elicit *pen* in monolinguals and *pencil* in bilinguals, possibly because of different collocation norms between the two languages (“pen and paper” in English vs. “papel y lápiz” in Spanish).

We also identified noteworthy patterns in participants’ responses that, while not directly tested statistically, may reflect linguistic and cultural influences on associative thinking that are worth investigating further in future work. First, several responses suggested that polysemes and homonyms—words with multiple meanings—may have served as “semantic bridges” between otherwise unrelated concepts. In Spanish, for example, *hoja* can mean “leaf” or “sheet of paper,” *tierra* can refer to “soil,” “land,” or “Earth,” and *vela* can mean both “candle” and “sail.” These types of words appeared to facilitate associations between semantically distant ideas (e.g., *papel* [paper] *→ hoja* [sheet/leaf] *→ árbol* [tree]; see Table 3 for more examples), illustrating how lexical ambiguity could draw together unrelated concepts. Second, we observed patterns that may reflect different behavioral and cultural norms between groups. Bilinguals, for example, were more likely than monolinguals to associate *paper* with school-related words (e.g., pencil, eraser, notebook) and *table* with food-related words (e.g., food, dinner, spoon). Monolinguals, on the other hand, were more likely to associate *paper* with work-related words (e.g., pen, ink, chair, office) and *table* with functional or structural words (e.g., leg, computer, lamp, seat). Finally, one monolingual participant achieved the highest Forward Flow score in a single trial (FF = .905) by producing several sequences of rhyming words (e.g., *table* → *fable* → *cable*, *stamp* → *camp* → *damp*), showing how phonological similarity could also shape associative links and lead to highly distant semantic trajectories.

**Table 3:** Linguistic and cultural features influencing word associations.

Feature	Example
Compounding	toaster → oven; candle → stick; paper → towel; table → cloth
Collocation	“pen and paper” (common in English) “papel y lápiz” (common in Spanish)
Polysemes and homonyms	papel (paper) → hoja (sheet / leaf) → árbol (tree) → tierra (soil / Earth) → planeta (planet) → espacio (space)
Culture	Bilinguals were more likely to associate *paper* with school-related words (e.g., pencil, eraser, notebook) and *table* with food-related words (e.g., food, dinner, spoon).
Phonology (rhyming)	**table → fable → cable → stable →** Naples → jaundice → candy → band → **tramp → stamp → camp → damp → amp** → electricity → conductor → tape → **steak → fake → rake**

## Discussion

The present study examined how language experience shapes free associative thought. Using the Forward Flow metric—a measure of how far a thought sequence diverges semantically from its initial concept—we examined associative thinking in English monolinguals and Spanish–English bilinguals. Forward Flow scores varied as a function of vocabulary and language dominance, with bilinguals showing higher Forward Flow in their dominant language compared to their non-dominant language. Follow-up analyses also revealed that the associations participants generated were systematically influenced by language-specific properties. Below, we discuss how word frequency, vocabulary, cultural norms, and certain linguistic features can shape the conceptual associations observed in the Forward Flow task.

Our finding that language experience influences Forward Flow adds to the emerging literature on associative thinking in bilinguals. Both Fernández-Fontecha and Kenett [12] and Yang et al. [13] found that dominance or higher proficiency in a language predicts higher Forward Flow scores, suggesting that bilinguals “travel further” in semantic space during free association in their more proficient language. Similarly, we observed that higher vocabulary and dominance predicted higher Forward Flow, but importantly, this effect was not limited to bilinguals. Among monolinguals, English vocabulary also predicted higher Forward Flow, indicating that greater language expertise facilitates the retrieval of more distant conceptual associations, a process closely tied to creative expression.

Although we did not explicitly test whether Forward Flow predicts creativity, our finding that vocabulary and language dominance modulate associative thinking mirrors the pattern observed in a previous study on verbal divergent thinking using the Alternate Uses Task. In that work, we found that bilinguals were more creative in their more-dominant language [37]. The findings of these two studies with bilinguals are consistent with monolingual evidence linking Forward Flow and performance on the Alternate Uses Task [5,6], suggesting that both tasks rely on similar associative processes. Bilinguals’ reduced *verbal* creativity in a less-dominant language may therefore stem from a constrained associative ability in that language, which is naturally limited by their vocabulary and ease of lexical access. In line with this, creative idea generation has been found to be strongly associated with broad retrieval ability and processing speed [38], both of which are reduced in a less proficient language [39]. Moreover, associative thinking in a non-dominant language may be further constrained by the increased cognitive demands of processing a less-proficient language [40,41], which may interfere with the retrieval of remote concepts. Beyond these psycholinguistic variables, socio-affective factors such as foreign language anxiety [42] and L2 motivation [43] have also been found to moderate creative performance in a non-dominant language. Taken together, these studies suggest bilinguals’ creative thinking in a less-dominant language may be impacted by a combination of linguistic, cognitive, and socio-affective influences.

The convergence between our Forward Flow results and prior Alternate Uses Task findings [37] may help explain the broader pattern of results in the bilingualism-creativity literature. A recent meta-analysis by Acar et al. found a small but reliable association between bilingualism and creativity [2]. Critically, however, this association was moderated by task modality: it was strongest for numerical creativity tasks and weakest for verbal creativity tasks [2]. This pattern is consistent with our own findings: proficiency and dominance influence both creative idea generation [37] and verbal associative thinking (current study). Bilinguals vary widely in proficiency, and because verbal tasks are naturally sensitive to this variation, studies that do not directly account for proficiency may show inconsistent results depending on the proficiency of the sample tested. This could help explain why verbal tasks show weaker and more variable effects than nonverbal tasks across the bilingualism-creativity literature. In this way, our findings help bridge two research areas that have largely developed independently: research showing that proficiency shapes the lexical associations bilinguals produce in each of their languages (e.g., Fitzpatrick and Izura [11]), and research linking associative thinking to creative performance [4].

When examining the linguistic variables associated with Forward Flow, we found that scores were highly influenced by the lexical properties of the generated words, particularly word frequency. Specifically, lower-frequency words were associated with higher Forward Flow scores, suggesting that using less common words elicited more semantically distant associations. This effect helps explain why participants with smaller vocabularies, such as bilinguals in their non-dominant language, showed reduced Forward Flow. Lexical frequency influences word access, with high-frequency words accessed more easily than low-frequency words [44,45]. Bilinguals are particularly sensitive to frequency effects as reduced language use in each language makes lower-frequency words harder to access and increases reliance on high-frequency words [46,47]. As a result, conceptual links in a less-dominant language may be constrained to more frequent, closely related concepts, limiting the semantic distance traveled during free association.

At the item level, these lexical constraints were reflected in differences across seed words. Lower-frequency seed words elicited more semantically distant associations (i.e., higher Forward Flow) than higher-frequency words, particularly in the beginning of the task (words two through six; see Fig 2). We likely saw this pattern because higher-frequency words tend to be more centrally located within a semantic network, forming dense, highly interconnected clusters [48]. As a result, starting with high-frequency words may promote access to more closely related concepts, leading to shorter semantic distances in association tasks. In contrast, lower-frequency words tend to occupy sparser and less connected regions of a semantic network, which may elicit more remote associations. Although our experiment used the same seed words as past studies [5,6,13], the item-level variation we found suggests that the semantic trajectory of associative thought is highly sensitive to lexical properties—an important consideration for future research using the Forward Flow metric.

These lexical effects further interacted with group language experience, as monolinguals and bilinguals showed distinct semantic trajectories that varied based on the given seed word. For example, following the lower-frequency seed *candle*, monolinguals produced specific, lower-frequency associations (e.g., flame, wick), resulting in a smaller, initial semantic distance but a steep increase over subsequent responses. Bilinguals, in contrast, were more likely to begin with high-frequency, general associations (e.g., fire, hot), yielding a larger initial semantic distance but a flatter overall trajectory. One explanation is that, compared to monolinguals, bilinguals tend to show reduced lexical access to low-frequency words [46,47]. As a result, lower-frequency words like *flame* and *wick* may not have been as accessible for bilinguals, leading them to use more high-frequency words like *fire* and *hot* (Fig 3). However, these differences could also reflect broader, language-level effects: although *flame* and *wick* are relatively low-frequency in English, their Spanish translations (*llama* and *mecha*) are even rarer, which may have further reduced their likelihood of being used.

Our analysis of the generated words revealed additional group-level differences that reflect deeper influences of language and culture. Monolinguals, for example, were more likely than bilinguals to produce the second element of English compound words or phrases (e.g., toaster *oven*, table*cloth*, paper *towel*). While compound words exist in both languages, noun-noun compounding is more common in English than in Spanish [49], which may have guided participants’ conceptual associations during the Forward Flow task. Similarly, certain generated items reflected language-specific collocations (i.e., words that commonly co-occur in a language). Monolinguals often followed the seed word *paper* with *pen* (consistent with the common English phrase *pen and paper*), whereas bilinguals were more likely to use *pencil* (*papel y lápiz* [paper and pencil] being the more common expression in Spanish). A Google Books Ngram Viewer query (2019, English and Spanish) for collocates of *paper* and *papel* [paper] supports this interpretation: *pen* was a more frequent collocate than *pencil* in English (0.55 vs. 0.30 per million trigrams), while *lápiz* [pencil] was a more frequent collocate than *bolígrafo* [pen] in Spanish (0.61 vs. 0.12 per million trigrams). These patterns illustrate how idiosyncratic linguistic features may shape the organization of semantic memory and the associative routes individuals take during free thought.

Beyond differences in linguistic features, our results also suggest culturally influenced patterns of association. Bilinguals were more likely than monolinguals to link *table* with meal-related words such as *food*, *dinner*, or *spoon*. These responses may reflect the cultural norm of eating meals together at the table, a practice that is more common among immigrant and Spanish-speaking families in the United States [50,51]. Taken together, the words generated by monolinguals and bilinguals suggest that associative thinking can be shaped not only by language and proficiency but also by the cultural experiences associated with each language group.

Finally, we found that word associations could be driven by phonological similarity. One monolingual participant generated several sequences of rhyming words (e.g., *table* → *fable* → *cable*, see Table 3), which resulted in the highest Forward Flow score of any given trial (FF = .905). By breaking away from purely semantic associations, this participant’s responses demonstrate how linguistic form (e.g., phonology) can shape associative thinking and yield more semantically distant thought sequences. This example illustrates the interaction of form and meaning, showing that associative thinking emerges from the interplay of multiple levels of language.

### Limitations and future directions

We examined the relationship between language and associative thinking, but our study has limitations for the generalizability of our findings. First, because bilinguals completed the free association task in both English and Spanish, they completed half as many trials per language as monolinguals, who completed all four trials only in English. Although the total number of English trials was comparable across groups given our larger bilingual sample size, monolinguals could have developed different or more varied strategies by having more trials in English. Future studies may want to equate the number of trials each group completes in each language or explore the strategies participants use trial-by-trial. Second, we explored the utility of Forward Flow as a measure to investigate the relationship between language and associative thought, using four seed words from the original Forward Flow validation study. Given the small number of starting seed words we used, our findings illustrate how language *can* shape word retrieval and concept association, but do not fully capture the complexity of bilingual lexical organization. To continue understanding the linguistic bases of associative thought, future studies will have to employ a wider range of seed words, use other free association tasks, and examine association in different language samples (e.g., speeches, essays, natural conversation).

The moderating effect of word frequency on the Forward Flow metric introduces a challenge in capturing associative thinking through verbal tasks, especially among multilinguals. As described previously, Forward Flow uses static word embeddings from distributional semantic models to calculate the semantic distance between words. For example, consider the high-frequency word sequence *water* → *moon* → *plant* and a parallel set with lower-frequency derivatives *aquatic* → *lunar* → *botanical*. While both sets vary in frequency, the meaning of words is conceptually similar and should theoretically reflect similar Forward Flow. However, when static word embeddings are used to compute semantic distances between word pairs, Forward Flow for the first set is 0.623 (a low Forward Flow score), whereas Forward Flow for the second set is 0.845 (a high Forward Flow score). This example illustrates how differences in word frequency can impact computationally derived semantic distances [52], and in turn, Forward Flow scores. To mitigate this influence, we included word frequency as a covariate in our analyses, but frequency norms derived from monolingual corpora (e.g., SUBTLEX-US [34], SUBTLEX-ESP [35]) may not fully capture the lexical representations of multilingual speakers. Importantly, the influence of word frequency is likely amplified for multilinguals who are more likely to use high-frequency words than monolinguals [46]. As a result, measures that rely on semantic distance (for a review, see [4]) may underestimate associative thinking in multilinguals, reflecting differences in lexical access rather than a true difference in concept association or creativity. To address this limitation, future research using large language models to study cognition will need to account for known psycholinguistic phenomena such as word frequency effects. More broadly, as large language models continue to evolve, their utility for understanding the human mind will be conditioned by the extent to which their symbolic representations can capture the variability of human language experience.

Another challenge of using language to probe conceptual associations stems from the inherent ambiguity of linguistic systems. Most word forms have multiple meanings that are usually related (e.g., polysemes), but can also be unrelated (e.g., homonyms [53]). In natural language use, a word’s multiple meanings can be disambiguated through context. In our free association task, however, participants were asked to generate single words, which limits context and thus obscures the meaning of the generated word. For example, the cue word *candle* often elicited the word “stick,” but it remains uncertain whether a participant’s intended meaning referred to a candlestick, a wooden stick, or an adhesive. Moreover, multilingual contexts requiring translation further complicate the issue of lexical ambiguity, since form-meaning mappings often do not align across languages (e.g., *hoja* in Spanish can refer to a sheet of paper or the leaf of a tree). Translation was a limitation of the current study: to score Forward Flow with the same model, we chose to translate Spanish responses into English. Although this approach allowed us to measure semantic distance and score Forward Flow consistently across languages, it comes at the cost of translations potentially not preserving the semantic relationships in participants’ original, untranslated responses. Future studies could examine how Forward Flow is scored across models and semantic spaces, or whether newer multilingual embedding models can reliably capture meaning across languages.

Modeling lexical ambiguity has been a longstanding challenge in both human [54,55] and natural language processing (NLP) research [56]. In NLP, work has focused on developing *word sense disambiguation* methods [57,58] to computationally discern word meanings. This challenge is directly relevant to our study, as computational estimates of semantic distance depend on a model’s underlying representations of meaning. The current implementation of Forward Flow estimates semantic distance using *static* word embeddings [24], which use a single vector per word and therefore cannot differentiate between multiple meanings of the same form. One potential solution would be to use more modern contextual embeddings [59], which can disambiguate word meanings based on context. However, how models trained on sentence-level corpora should be used to estimate distances between isolated words is unclear. Others have tackled lexical ambiguity by fine-tuning models to produce *word sense embeddings* [60] or *semantic word embeddings* [61] that can capture distinct senses of polysemous words. Ultimately, accurately measuring conceptual associations through verbal responses will require methods that account for linguistic ambiguity.

## Conclusion

Our findings suggest that the conceptual associations individuals make during free associative thought are linked to language experience and features of the languages they speak. Greater vocabulary and language dominance predicted more semantically distant thought sequences, suggesting that language expertise may facilitate remote conceptual associations. Associative thinking was also influenced by linguistic properties like word frequency and lexical ambiguity, which shaped the semantic trajectory of individuals’ thoughts. Because association serves as a cognitive foundation for creativity, these language-related differences in associative thinking may cascade into broader differences in creative thought. We conclude that to advance our understanding of human cognition, future research will need to integrate emerging computational approaches with theories of multilingualism, embracing the complexities of multiple interacting linguistic systems.

## Supporting information

S1 File Supporting file containing supplementary tables S1-S3. Likelihood ratio tests for fixed effects predicting Forward Flow (Table S1); Pairwise comparisons of IFF means for first generated word (Table S2); Pairwise comparisons of IFF slope for first five generated words (Table S3).
(DOCX)
