# Supplementary material for: Free flowing thought in bilinguals and monolinguals: How language experience and lexical properties influence word associations
Source: PLoS One. 2026 Oct 5;21(10):e0359889. doi: 10.1371/journal.pone.0359889 (PMC13637863; doi:10.1371/journal.pone.0359889)
Supplement: Supplementary file 1 [file pone.0359889.s001.docx]

Supporting information for

Free flowing thought in bilinguals and monolinguals:

How language experience and lexical properties influence word associations

Matias Fernandez-Duque^1^ and Viorica Marian^1,2^

^1^Department of Communication Sciences and Disorders, Northwestern University, Evanston, United States

^2^Department of Psychology, Northwestern University, Evanston, United States

**Table S1. Likelihood ratio tests (LRTs) for fixed effects predicting Forward Flow (FF).**

| Predictor | df | *χ^2^* | *p*-value |
| --- | --- | --- | --- |
| Task Language Dominance | 2 | 17.64 | < .001 |
| English Vocabulary | 1 | 1.00 | 0.316 |
| Seed Word | 3 | 35.10 | < .001 |
| Word Frequency | 1 | 275.02 | < .001 |
| Language Group × English Vocabulary | 2 | 6.54 | 0.038 |
| Word Frequency × Seed Word | 3 | 28.05 | < .001 |
| Model: Forward Flow ~ TaskLangDom*EngVocab + SeedWord*WordFreq + (1 + Trial \| Subject) | | | |

**Table S2. Pairwise comparisons of IFF means for first generated word.**

| Pairwise comparison | Estimate | SE | t.ratio | p.value |
| --- | --- | --- | --- | --- |
| candle - paper | 0.117 | 0.007 | 17.365 | < .001 |
| candle - table | 0.087 | 0.007 | 13.021 | < .001 |
| candle - toaster | -0.023 | 0.006 | -3.731 | 0.001 |
| paper - table | -0.030 | 0.007 | -4.174 | < .001 |
| paper - toaster | -0.140 | 0.007 | -21.300 | < .001 |
| table - toaster | -0.110 | 0.007 | -16.866 | < .001 |

*Note*. Estimate = difference in estimated marginal mean between the two seed words listed in each pairwise comparison (first word minus second word).

**Table S3. Pairwise comparisons of IFF slope for first five generated words.**

| Pairwise comparison | Estimate | SE | t.ratio | p.value |
| --- | --- | --- | --- | --- |
| Seed Word = candle |  |  |  |  |
| Monolingual - Bilingual (English) | 0.017 | 0.003 | 5.207 | < .001 |
| Monolingual - Bilingual (Spanish) | 0.023 | 0.003 | 7.304 | < .001 |
| Bilingual (English) - Bilingual (Spanish) | 0.005 | 0.002 | 2.192 | 0.073 |
| Seed Word = paper |  |  |  |  |
| Monolingual - Bilingual (English) | -0.006 | 0.004 | -1.548 | 0.269 |
| Monolingual - Bilingual (Spanish) | -0.008 | 0.004 | -2.101 | 0.090 |
| Bilingual (English) - Bilingual (Spanish) | -0.002 | 0.004 | -0.540 | 0.852 |
| Seed Word = table |  |  |  |  |
| Monolingual - Bilingual (English) | 0.003 | 0.004 | 0.923 | 0.626 |
| Monolingual - Bilingual (Spanish) | 0.005 | 0.004 | 1.447 | 0.317 |
| Bilingual (English) - Bilingual (Spanish) | 0.002 | 0.004 | 0.545 | 0.849 |
| Seed Word = toaster |  |  |  |  |
| Monolingual - Bilingual (English) | -0.002 | 0.003 | -0.877 | 0.655 |
| Monolingual - Bilingual (Spanish) | 0.003 | 0.002 | 1.470 | 0.306 |
| Bilingual (English) - Bilingual (Spanish) | 0.006 | 0.003 | 2.200 | 0.071 |

*Note*. Estimate = difference in IFF slope between the two groups listed in each pairwise comparison; positive values indicate a steeper slope for the group listed first.
